# A qualitative study of women’s views on overdiagnosis and screening for thyroid cancer in Korea

**DOI:** 10.1186/s12885-015-1877-6

**Published:** 2015-11-06

**Authors:** Sang Hee Park, Bomyee Lee, Sangeun Lee, Eunji Choi, Eun-Bi Choi, Jisu Yoo, Jae Kwan Jun, Kui Son Choi

**Affiliations:** 1 Graduate School of Communication, Sogang University, 35 Baekbeom-ro, Mapo-gu, Seoul, 121-742 Republic of Korea; 2 National Cancer Control Institute, National Cancer Centre, 323, Ilsan-ro, Ilsandong-gu, Goyang-si, Gyeonggi-do 410-769 Republic of Korea; 3 Graduate School of Cancer Science and Policy, National Cancer Centre, 323, Ilsan-ro, Ilsandong-gu, Goyang-si, Gyeonggi-do 410-769 Republic of Korea

**Keywords:** Thyroid cancer, Overdiagnosis, Screening, Qualitative

## Abstract

**Background:**

The incidence of thyroid cancer in Korea has increased by about 25 % every year for the past 10 years. This increase is largely due to a rising incidence in papillary thyroid cancer, which is associated with an overdiagnosis of small tumors that may never become clinically significant. This study was conducted to explore Korean women’s understanding of overdiagnosis and to investigate changes in screening intention in response to overdiagnosis information.

**Methods:**

Focus group interviews were conducted among women of ages 30–69 years, who are commonly targeted in Korea for cancer screening. Women were divided into four groups according to thyroid cancer screening history and history of thyroid disease. Of 51 women who were contacted, 29 (57 %) participated in the interviews.

**Results:**

Prior awareness of thyroid cancer overdiagnosis was minimal. When informed about the risks of overdiagnosis, the participants were often surprised. Overcoming initial malcontent, many women remained skeptic about overdiagnosis and trusted in the advice of their physicians. Meanwhile, some of the study participants found explanations of overdiagnosis difficult to understand. Further, hearing about the risks of overdiagnosis had limited impact on the participants’ attitudes and intentions to undergo thyroid cancer screening, as many women expressed willingness to undergoing continued screening in the future.

**Conclusion:**

A large majority of Korean women eligible for and had undergone thyroid cancer screening were unaware of the potential for overdiagnosis. Nevertheless, overdiagnosis information generally had little impact on their beliefs about thyroid cancer screening and their intentions to undergo future screening. Further research is needed to determine whether these findings could be generalized to the wider Korean population.

## Background

Over the last 10 years, the incidence of thyroid cancer in Korea has increased every year by about 25 %, such that thyroid cancer now leads as the most common type of cancer in women across the nation [1]. In fact, more than 40,000 people were diagnosed with the disease in 2011. More specifically, the proportion of small (<1 cm) tumors detected has increased significantly from 6 % in 1962 to 43 % in 2009; meanwhile, mortality rates for thyroid cancer have remained constant [2]. According to estimates by GLOBOCAN 2012, the incidence of thyroid cancer among Korea women (age standardized rate, 88.6 per 100,000 population) is more than 18 times greater than that for the UK and 4.4 times that for the US [3].

This increase in the incidence of thyroid cancer in Korea is largely due to a rising incidence in papillary thyroid cancer (PTC), which is associated with an excellent prognosis and a 10-year survival rate of 98.6 % [4]. PTC is the most common subtype of thyroid cancer, accounting for 90 % of all thyroid cancer occurrences in Korea, and its incidence has increased annually by 25.1 % in men and 23.7 % in women from 1997 through 2011 [5]. Nevertheless, despite favorable long-term survival, PTC is associated with significant morbidity and societal burden. While studies have yet to outline definitive reasons for increases in thyroid cancer incidence, some have suggested that the apparent increases in thyroid cancer do not represent a true increase in disease but rather result from a large reservoir of undiagnosed PTC combined with overdiagnosis of small tumors that will never become clinically significant [6–8].

Although thyroid-cancer screening is not included in the National Cancer Screening Program in Korea, healthcare providers frequently choose to offer opportunistic screening for thyroid cancer with ultrasonography as an inexpensive add-on for about 30 to $50. Moreover, the offices of many general practitioners are equipped with ultrasonography machines for which to scan the thyroid for cancer. Interestingly, a previous report in Korea noted a strong correlation between thyroid cancer screening rates and the incidence of thyroid cancer by region [6].

While generally positive, screening for the early detection of cancer can lead to overdiagnosis, resulting in the overtreatment of inconsequential disease and thereby unwarranted physical and emotional harm [9]. Despite the harms of overdiagnosis, little is known about the public’s understanding of overdiagnosis or how overdiagnosis information may affect one’s decision to undergo screening. Notwithstanding, recent qualitative studies found that information on overdiagnosis was difficult for screening-eligible women to understand and was often surprising [10, 11].

Thus, we conducted a qualitative study to assess responses to information about overdiagnosis in thyroid screening among Korean women. The study was designed to highlight their understanding of information on overdiagnosis, as well as to investigate changes in their intentions to undergo screening in the future in response to overdiagnosis information.

## Methods

### Study design and setting

In the present study, we conducted focus group interviews to explore existing knowledge of and experiences with thyroid cancer screening among Korean women. We also attempted to discover how women integrated information about overdiagnosis with their existing understanding of thyroid cancer screening and how they might utilize the information when deciding whether or not to undergo continued screening. Collecting data through focus groups, rather than individual interviews, allowed participants to raise and discuss relevant points beyond those anticipated by the researchers [12]. All participants provided written consent prior to participation in the interviews. This study was approved by the Institutional Review Board of the National Cancer Center, Korea (IRB No.: NCCNCS08129).

### Recruitment

We conducted four focus group interviews with women of ages 30–69 years, who are commonly targeted for cancer screening. In order to ensure the homogeneity within each group, we included women who had the same experiences with thyroid cancer screening. Based on their histories of thyroid cancer screening and thyroid disease, the following four groups were formed: Group 1, women who had never undergone thyroid cancer screening; Group 2, women who had undergone thyroid cancer screening (ultrasonography test) within the last 24 months with negative results; Group 3, women with benign thyroid nodules, for which they visited the hospital regularly for a check-up; and Group 4, women who underwent a thyroidectomy to remove all or part of the thyroid gland after being diagnosed with thyroid cancer. While each individual focus group was characteristically homogeneous, the differences between the groups were large enough to allow for contrasting opinions.

Participants were recruited at the Center for Cancer Prevention & Detection with the National Cancer Center, Korea via advertisements posted throughout the National Cancer Center Hospital. The advertisement described our search for women who would be willing to participate in a focus group interview to discuss overdiagnosis and thyroid cancer screening, along with information about the purpose of the study, the interview duration, selection criteria, and a financial incentive (about 70 US dollars) for participation. Among volunteers who responded to the advertisement, we primarily looked for women who fulfilled criteria for inclusion in one of the four groups. We then chose prospective participants after considering the age distribution of each group and on a first-come, first-served basis. The focus group sessions were scheduled to best accommodate the availability of the participants; we proposed four alternate interview days to 51 women who were finally contacted. A total of 29 (57 %) participated in the interview. In this study, each group comprised five to ten women: typically, the ideal size of a focus group for most noncommercial topics is five to eight participants [13].

### Data collection

The interviews were conducted in August 2014 in a conference room at the National Cancer Center, Korea, and were facilitated by an experienced qualitative researcher, with a second researcher acting as an observer. To ensure data authenticity, we selected a neutral “third party” interviewer to conduct the interviews. The interviewer was given our expectations for the study, preconceptions, values, and orientation information, including any theoretical commitments. Also, to prevent questions from being asked in a way that may lead the participants to answer in a particular manner, we provided the interviewer an interview guide (Table 1). In designing the discussion guide, emphasis was placed on constructing open-ended, non-directive questions and using a funneling approach, with questions moving from the general to the more focused.

**Table 1:** Focus group interview guide and key discussion questions

ㆍSelf-assessment of health and health behavior including cancer screening ㆍOverall knowledge of cancer, cancer prevention, and screening ㆍKnowledge of thyroid cancer and thyroid cancer screening ㆍPerceptions of overdiagnosis ㆍProvide information on thyroid cancer screening and overdiagnosis	
	-Thyroid cancer screening incidence, mortality, and survival rates in Korea: incidence has increased 15-fold over the past two decades; the most commonly diagnosed cancer in women. Almost all newly identified thyroid cancers are tiny papillary thyroid tumors (very slow-growing, highly unlikely to go on to cause symptoms, and much less death). Mortality rates have not budged over the past 20 years; 5-year relative survival rate is almost 100 %.
	-Introduction to thyroid cancer screening: test with ultrasonography costs 30 to 50 USD
	-Definition of overdiagnosis: diagnosis of thyroid cancer that may not go on to cause symptoms or death in your life time
	-Harms of overdiagnosis: unnecessary effects of thyroidectomy: a lifelong calcium-metabolism condition; vocal cord paralysis, etc.
ㆍPros and cons of thyroid cancer screening and changes in intentions to undergo screening in the future ㆍInformation needs for thyroid cancer screening	

Before starting the focus group discussion, participants were asked to complete a short questionnaire assessing sociodemographic characteristics (age, education, and income level), family history of cancer, and previous history of thyroid cancer screening among family members. To open the discussion, in accordance with the focus group interview guide (Table 1), participants were invited to discuss their own experiences with deciding whether or not to undergo thyroid cancer screening. They were then asked to read information concerning thyroid cancer screening, which included a statement on overdiagnosis (“Screening can discover treatable cancers that may have otherwise gone unnoticed during your lifetime.”) After briefly reading aloud additional information on overdiagnosis, women were asked to discuss how this information might affect their views of thyroid cancer screening. Group discussions were digitally recorded and transcribed verbatim.

### Analysis

In the present study, we conducted a thematic analysis, aiming to identify a set of main themes that captured the diverse views and feelings expressed by the participants. Verbatim transcripts were analyzed thematically. Two researchers read all of the transcripts independently and generated initial codes. These were then collated into potential themes. Using constant comparison, we strived to highlight similarities and differences in the data, as well as coding within and across transcripts. Meeting regularly to discuss the framework with which to interpret the data, we compared parts of the data with the rest as a whole, establishing analytical categories and selecting key themes. In this study, two researchers coded a total of 812 verbatim transcripts. The two researchers finally selected 40 transcripts to be coded for inclusion in the framework. Among these 40 transcripts, 33 were given the same codes by the two researchers. Inter-coder reliability was 0.825 (Holsti index).

## Results

### Participant characteristics

The demographic characteristics of all 29 individuals who agreed to participate in this study are described in Table 2. Among the participants, those in their 50s were most common, followed by those in their 30, 40, and 60s. According to household income, 40 % of participants in Group 1, those who had never undergone thyroid cancer screening, earned less than 2000 US dollars per month. While the proportion of those who responded that they knew of a family member or acquaintance who had thyroid cancer was high for the entire study population, Group 1 participants more often responded that they did not know of anyone close to them with thyroid cancer.

**Table 2:** Characteristics of the participants

	Group 1	Group 2	Group 3	Group 4	Total
	(*n* = 10)	(*n* = 7)	(*n* = 5)	(*n* = 7)	(*n* = 29)
Age (years)					
30–39	2 (20.0)	3 (42.8)	1 (20.0)	3 (42.8)	9 (31.0)
40–49	3 (30.0)	1 (14.3)	2 (40.0)	1 (14.3)	7 (24.1)
50–59	3 (30.0)	3 (42.8)	2 (40.0)	2 (28.6)	10 (34.5)
60–69	2 (20.0)	-	-	1 (14.3)	3 (10.3)
Monthly household income (US $)					
≤1,999	4 (40.0)	-	-	2 (28.6)	6 (20.7)
2,000–3,999	3 (30.0)	-	3 (60.0)	2 (28.6)	8 (27.6)
4,000–6,999	2 (20.0)	5 (71.4)	1 (20.0)	2 (28.6)	10 (34.5)
≥7,000	1 (10.0)	2 (28.6)	1 (20.0)	1 (14.3)	5 (17.2)
Family history of thyroid cancer (or a friend diagnosed with thyroid cancer)					
Yes	4 (40.0)	6 (85.7)	3 (60.0)	6 (85.7)	19 (65.5)
No	6 (60.0)	1 (14.3)	2 (40.0)	1 (14.3)	10 (34.5)

### Knowledge of thyroid cancer and thyroid cancer screening

When asked about thyroid cancer and thyroid cancer screening, as well as where they received their information thereon, the majority of the participant responded saying they thought thyroid cancer is a slowly developing cancer with a higher survival rate, and thus, did not regard it as serious. This perception was especially common among Group 1 and Group 2 participants: 81 % recognized thyroid cancer as a non-fatal cancer, whereas 15 % of women in Group 3 and Group 4 thought it was likely to develop into severe disease and to negatively affect their quality of life.

*“While I have seen people with thyroid cancer, I heard that it grows slowly and that it can be cured without difficulty, if it is found early enough. I do not take it to be serious.”*

*“I only know that the majority of thyroid cancer patients are women and that Korea has a higher incidence thereof.”*

Meanwhile, many of the participants reported knowing very little about the function and location of the thyroid, as well as risk factors for thyroid cancer, particularly participants in Groups 1 and 2. Only 18 % of women in Group 1 and Group 2 who had a thyroid cancer patient in their family knew the function and location of the thyroid, as well as risk factors for thyroid cancer. However, 100 % of women in Group 3 and Group 4 knew extensive information about thyroid cancer, except for the adverse effects of thyroid surgery.

*“I have no idea where the thyroid is or what function it serves.” (Group 2 participant)*

*“I suspect that thyroid cancer may result from improper self-management, such as bad habits or a lack of exercise.” (Group 2 participant)*

*“Since the thyroid controls the hormone system, I would guess that stress might be the cause [of thyroid cancer], but I do not know very well.” (Group 1 participant)*

Regarding where the participants had received their information on thyroid screening, 51 % of participants reported hearing about it from the media (e.g., internet and TV); however, none responded that they learned about it from their doctors. Group 1 participants typically reported knowing nothing about thyroid cancer screening procedures. Many of the participants who had experiences with screening stated they had received information about thyroid cancer screening during routine health check-ups; their motivations to undergo screening primarily stemmed from a family history of cancer (10 % of the participants) or recommendations from close acquaintances (10 % of the participants), rarely from observable symptoms (3 % of the participants).

### Perceptions of overdiagnosis

During the interviews, we particularly noted controversy among the participants regarding the potential dangers of overdiagnosis in thyroid cancer screening. We have outlined the participants’ reactions to the following statement regarding overdiagnosis in thyroid cancer: “Recently, cases of thyroid cancer are rapidly increasing, largely due to overdiagnosis from cancer screening. Overdiagnosis is the diagnosis of disease that will never cause symptoms or death during a patient’s lifetime, and may lead to treatments that are of no benefit and perhaps harmful.”

The participants’ reactions to this statement were summarized into five main types: confusion, denial, malcontent, trust in physicians, and indifference.

- Confusion: The majority of the participants (90 %) stated that did not entirely grasp the meaning of overdiagnosis, and 15 % of them responded that the concept thereof was difficult to comprehend. All of the participants in Groups 1 and 2 expressed such confusion.

*“I have never heard about overdiagnosis. Despite learning about it in this interview, I still do not fully understand it.”*

*“On TV, many programs talk about whether or not one should undergo thyroid screening, seek treatment, etc. The differing opinions are very confusing and make it harder to understand what to do.”*

*“For the elderly, it [overdiagnosis] is difficult to understand and complicates making a decision.”*

(b) Denial: Some of the participants (17 %) considered thyroid cancer as a major cancer type, and they denied overdiagnosis as a serious issue. These participants expressed a strong belief that cancer screening for preventive purposes could not be adverse to one’s health. This was particular apparent among Group 3 participants, those with benign thyroid nodules, who communicated complete trust in their doctors to watch over their health.

*“The word ‘overdiagnosis’ is inappropriate. I think finding disease early and seeking timely treatment is very important. [We can ensure our health through screening.]”*

*“For preventive purposes, early discovery of disease is necessary, though it may cause overdiagnosis.”*

*“People have stated that thyroid cancer is unnecessarily overdiagnosed; however, I do not agree. Every single disease must be identified. Whether or not one should be treated is up to the individual, but just finding disease is not unreasonable.”*

*“Is it not natural to seek treatment or surgery if there is a defect? I do not think it is too excessive.”*

*“I heard that the National Health Insurance Service plans to cut reimbursement for thyroid cancer diagnostic and treatment tests to save money. I think that is why the media frequently negatively reports on overdiagnosis from thyroid cancer screening. This whole thing is a conspiracy”*

(c) Malcontent: Overall 15 % of women voiced displeasure with healthcare professionals concerning a lack of information on overdiagnosis of thyroid cancer. Those who had undergone screening were surprised and annoyed that they were not informed about overdiagnosis prior to screening and treatment. Additionally, women were generally more worried about overtreatment than overdiagnosis. Nearly 10 % of women even suspected that physicians may encourage thyroid cancer screening for their own financial benefit.

*“I have heard about overdiagnosis. I heard it from my acquaintances, also from the internet. Screening, in my opinion, might be okay, although it is actually excessive, but overtreatment is not. I have read that the survival rate for thyroid cancer is nearly 100 %, so its treatment might be quite simple, making thyroidectomy excessive. I suspect that the main reason why doctors perform [complete] thyroidectomy is because they lack the surgical skills needed to preserve the thyroid. I will continue to undergo screening, but seeking treatment is another issue. Should any symptoms become serious, I would then seek treatment; otherwise, I would want to consider other options to manage it.”*

*“Ten years ago, my doctors recommended a total thyroidectomy to prevent metastasis, but now I regret. I was not able to get information through internet at that time. Now, I think the surgery was unwarranted, and I regret it.”*

*“Doctors recklessly provide thyroid cancer surgery for their own financial benefit. My sisters, who have already been diagnosed with thyroid cancer, do not recommend thyroid cancer screening.”*

*“I want doctors to set guidelines through discussion. It is time that they express sincerity.”*

(d) Trust in physicians: Although some participants expressed confusion about overdiagnosis, 27 % of women still conveyed trust in their doctors. The participants believed that their physicians would rightly determine whether thyroid cancer screening was needed or not, because they are experts. This was particular apparent among Group 3 participants.

*“I do not want to be suspicious of doctors who screen and treat disease.”*

*“The press tends to exaggerate facts. We need to trust doctors and their decisions.”*

*“Doctors know more about disease than laypeople. So, if screening is recommended by my doctor, then I will undergo the test.”*

(e) Indifference: Overall, 14 % of the participants showed a lack of concern for thyroid cancer and overdiagnosis. This was particular apparent among Group 1 and Group 2 women.

*“I have come across related material on TV, but I just skip it because I have no interest in it.”*

*“I have not heard and do not know anything about these. I haven’t even tried to get any information on them.”*

### Impact on screening decisions

After providing the information of overdiagnosis, we asked whether the participants would continue to undergo thyroid cancer screening in the future or not. While responses varied, 93 % of the participants were willing to adhere to screening. Participants who were already diagnosed with thyroid cancer or who were undergoing regular check-ups for their benign nodules responded that they would continue to undergo screening and would recommend it to others.

“*Definitely, I will continue to undergo thyroid cancer screening. The cost of screening is inexpensive. Because all diseases have a possibility to become a threat to my life, I myself will go on with thyroid cancer screening, regardless of the debate surrounding it.”*

*“Overtreatment is a problem, but not overdiagnosis. I will just go ahead with screening.”*

*“Screening should be continued. Thyroid cancer is still a cancer, and screening is required for prevention, no matter how big or small the cancer is.”*

*“As one who has been diagnosed with thyroid cancer, I feel that screening is necessary. I heard it during screening examination, and I also agree, that participating in screening is better, although thyroid cancer is slow growing, because thyroid cancer rapidly spreads in those of younger age.”*

*“I think my choice to be screened was right, and if asked, I would recommend it to others.”*

Meanwhile, changing their intentions to undergo screening, 7 % of women in Group 2 responded that the harms of thyroid cancer screening due to overdiagnosis seem to be greater than the benefits thereof.

“*No, I do not want to get thyroid cancer screening. There is no need to screen for thyroid cancer at all. I only need to get screening if I have symptoms; otherwise, I do not want to do it.*”

As well, women tended to rely strongly on their physicians’ decisions, although they expressed a willingness to lengthen the screening interval after consulting with their physician.

*“Every year when I undergo screening, I feel uncertain. My doctor does not talk about quitting the screening though… I would feel better if doctors would tell me that it is okay to be screened every two or three years.”*

*“It would be better if doctors specified when we should start screening and follow-up intervals thereafter. There should be clearer guidelines for thyroid cancer screening.”*

*“I will not undergo screening every year, if my doctor agrees. If there were guidelines, I would comply with them. Actually, I am supposed to get screened this year, but now I have no intention to after hearing about overdiagnosis today.”*

### Implications of overdiagnosis information

Herein, reactions to information on overdiagnosis differed among the participants according to group. Participants in Group 4, who had received a thyroidectomy after being diagnosed with thyroid cancer, were highly interested in the issue of overdiagnosis, and generally, despite of the possibility of overdiagnosis, felt that they had received timely and proper surgery. They added, however, that they were not fully aware of the side effects of the surgery, and felt disappointment in that they were not informed about overdiagnosis prior to surgery.

*“After my surgery, I gained more than 30 kg, and my nails and hair are not the same as before. The huge scar left by the surgery is also stressful. I often have to go on business trips, and whenever I go abroad, I need to get up and take pills at the specified time, which is annoying. Had I known about overdiagnosis and the side effects of the surgery, I would have thought about other options. Sufficient and accurate information about thyroid cancer and the side effects of surgery should be provided.”*

While women in Groups 2 and 3 remained confused about overdiagnosis, they still expressed a desire to have access to more detailed information thereon to help guide their decisions. Group 1 participants exhibited the lowest interest in overdiagnosis and the highest percentage of those who responded that it was their first time to hear about overdiagnosis. Confused about overdiagnosis information, the women in Group 1 suggested that national guidelines on an appropriate age and interval for thyroid cancer screening should be brought forward, as in guidelines for other types of cancer. They voiced hope that such guidelines would become available to help guide their decisions to undergo screening in the future.

*“Doctors should give and share well-documented information with patients, so that laypeople might be able to make an informed decision.”*

## Discussion

In this qualitative study, we noticed a lack of awareness about the risks of overdiagnosis in thyroid cancer screening among the participants. Participants were often shocked by information on overdiagnosis. Surprisingly, many women expressed skepticism about the harms of overdiagnosis, and most trusted that their physicians would make an appropriate decision on the necessity of screening tests. Meanwhile, some women suspected that doctors only offered thyroid cancer screening and treatments for their own financial benefit.

Although nearly all of the participants were surprised by information on overdiagnosis, its impact on the women’s attitudes toward screening varied. Nevertheless, most women were unaffected by learning about the possibility of overdiagnosis and expressed a willingness to undergo continued screening. Some, however, stated that this information would definitely affect their decisions to undergo screening in the future. These differences in screening intentions seemed to be related with the women’s previous history of thyroid cancer screening. Women who had previously undergone thyroid cancer screening were more likely to maintain a positive attitude toward thyroid cancer screening than those who had not. They particularly indicated that they were more afraid of getting cancer than overdiagnosis or overtreatment. Nonetheless, concerned with the information provided, a few of these women communicated a desire to lengthen the interval between screenings and felt that overdiagnosis might deter them from attending future screening all together. Despite differences in the way that information on overdiagnosis was presented, our findings are similar to those of a recent breast cancer screening study [10, 11], and suggest that similar challenges are faced among countries where screening is offered.

The perspectives of the women who participated in this study highlight three important insights into awareness of overdiagnosis in thyroid cancer screening. First, our participants sometimes found it difficult to understand the brief explanation of overdiagnosis that we provided, suggesting a need for better ways to communicate the importance and risks of overdiagnosis. In the current study, women seemed to be better understand the term “overtreatment” than “overdiagnosis.” A recent Australian study also reported that women were more likely to use “overtreatment” than “overdetection” [10]. Additionally, as further evidence of confusion concerning overdiagnosis in the present study, many women felt that screening was extremely important for the early detection and treatment of cancer, regardless of its likelihood of becoming malignant. In similar context, one study proposed that some pre-malignant conditions should not be labeled as cancers or neoplasia [14]. The authors suggested that use of the term “cancer” should be reserved for describing lesions with a reasonable likelihood of lethal progression if left untreated. Further, the other study also recommended that better predictive classification of tumours is needed in order to avoid unnecessary cancer diagnoses and subsequent procedures [15]. Nonetheless, further research is needed to identify which ways would best help lay individuals conceptualize overdiagnosis.

Second, many women indicated that further information on overdiagnosis would likely not change their intentions to undergo future screening. In general, women’s attitudes toward cancer screening are shaped through the information presented to them directly by screening service providers, as well as their broader experiences with public health campaigns that promote the benefits of screening without explaining the harms of overdiagnosis [16, 17]. Thus, one would expect that new information confounding this message (i.e., information about overdiagnosis) would not be immediately accepted and understood, as seen in our study. Accordingly, providing balanced information on the effectiveness of cancer screening and cultivating increased awareness of the harms thereof are needed to overcome current misplaced perceptions of the importance of cancer screening among the Korean public.

Finally, women in the present study reported that the recommendations of physicians were most likely to influence their screening behavior. Although some women showed distrust of physicians after hearing about information of overdiagnosis, the majority of women wanted to discuss this issue with their physician. In general, the female participants believed that their physicians were best able to determine whether thyroid cancer screening was needed or not, because of their expertise. However, in Korea, women are typically invited to undergo screening directly by screening centers and may attend screening without discussing the necessity thereof with their personal physician. In fact, the majority of the participants in the current study said that they received no explanation of the benefits and harms of thyroid cancer screening before they underwent the test.

This study involves a few limitations that warrant consideration. As the study sample was not designed to be statistically representative, we cannot conclude that our findings reflect the views of the general population. Further, individuals who were not included in the study may have different characteristics from those who participated in the focus group interviews. This can be a potential source of selection bias. Thus, larger population-based studies are needed to help elucidate female perspectives on overdiagnosis in order to develop more effective strategies with which to convey pertinent information.

## Conclusion

Although early detection of cancer is proven to save lives, in some instances it can be harmful, such as those related with overdiagnosis. In this study, we noticed difficulties among women who participated in this study with understanding the concept of overdiagnosis. Herein, overdiagnosis information itself had little impact on their intentions to undergo future screening. Additionally, women in the present study showed a strong reliance on their physicians’ recommendations, and wanted to further discuss issues regarding overdiagnosis with their physician. More work is needed to discover whether our findings could be generalized to the wider Korean population and to develop effective ways to communicate information on overdiagnosis.

## Abbreviation

PTC: Papillary thyroid cancer
